# Intermittent Fever, Hepatitis, Pneumonitis, Laryngitis and Intestinal Mucous Irritation

**Published:** 1840-11

**Authors:** Daniel Drake

**Affiliations:** Professor of Clinical Medicine and Pathological Anatomy in the Medical Institute of Louisville


Art. IV.—*Intermittent Fever, Hepatitis, Pneumonitis, Laryngitis and Intestinal Mucous Irritation.* By Daniel Drake, M. D., Professor of Clinical Medicine and Pathological Anatomy in the Medical Institute of Louisville.

We have written down these words to draw the reader's attention to a case which this day fell under our observation. We do not claim for it any *very* remarkable character, but as it is perhaps equal in interest to much that is published in medical journals we venture to send it forth.

Edward Dykes, a laboring man, living in the valley of Salt Creek, Chillicothe, Ohio, aged 32, of a sanguine temperament, and free from tubercular or other hereditary taint, was attacked about sixteen years ago, in autumn, with ague and fever, which lasted two months. For the next fourteen years it returned almost every atumn. During that long period, he continued most of the year to labor, and was free from cough, but sometimes had irritable bowels with slight diarrhœa.

In August, 1838, he was seized with the malady which had so often attacked him before. The type was at first tertian, and the cold stage amounted to a shake; at length became quotidian, with a chill only, about six o'clock, P. M. He continued to go about. Living with a "steam-doctor," he took two profuse sweats, and after the second "caught cold," and began to cough; at first without pain, but at length that symptom set in—being "located" in the right hypochondrium. His expectoration was sparing. He took two vomits of ipecac. but was neither bled nor blistered. Throughout the whole of the following winter, 1838, '39 he continued to have an evening chill, followed by fever in the early, and sweats in the latter part of the night, and frequent attacks of diarrhœa. His cough still continued with little expectoration. His larynx became somewhat sore and his voice and cough hoarse and flat, which sounds have continued ever since. Much eating brought on fits of coughing, and these brought up, by exciting a retrograde œsophageal action, the food which oppressed his stomach. Throughout the whole of the year 1839, these symptoms, including the evening chill, continued to return upon him, but he still kept on his feet, and did not become very much emaciated. In the month of January last, 1840, he awoke in the morning with severe pain extending from the clavicle to the short ribs on the right side, with sense of suffocation, and a new reduction of the powers of his voice. He was bled, cupped, blistered and took expectorants; under which the symptoms rapidly abated. Since that time he has not had evening chills, nor morning sweats; though he thinks his feet and hands hot at night; but his diarrhœa has continued. He has from five to eight evacuations daily. The discharges in the forenoon, often appearing to have received a tinge from the food he had taken the day before, but frequently of a light hue. In the afternoon they are small in quantity and attended with tenesmus, and very commonly have the appearance of "matter from a sore," which has been the case for eighteen months. His urine is often saffron colored. His cough is slight and his expectoration extremely limited. Twice or thrice lately the mucus has been tinged with blood.

At this time he has a moderately furred tongue, natural in size and color. His eyes although a little turbid, are not jaundiced. His skin generally is reduced in whiteness, but that of his face is dark and sallow, nearly up to his hair. The ends or balls of his fingers are a little enlarged. His pulse is 76, quiet and regular—not hard.

*Examination of the Neck and Chest.*—LARYNX with a tender spot on the right side, within the thyroid cartilage. Os hyoides exceedingly moveable. A grating sound when the larynx is moved by the hand. CHEST, when examined, naked and in quiet respiration, well formed, except a little bulging out of the most convex part of the false ribs, of the right side; which side, however, has the same circuit with the left both there and above. Breathing chiefly diaphragmatic. When directed to make a deep inspiration, his left shoulder ascends and the ribs rise and bulge out; but the right remains unmoved. When a cord was passed round the body, with its middle pressed upon one of the spinous processes, and the ends were brought to the sternum, a deep breath rendered the last extremity too short, but there was no recession of the right end. Under percussion no hollowness was discovered over any part of the right lung or of the *sternum*, either in the erect or recumbent position—the hollow sound of the left side was rather greater than common. Auscultation could detect no respiratory murmur, anywhere over the right side; but disclosed a considerable degree of broncophony with an occasional sibilant ronchus. His respiration throughout the left lung was puerile.

The position and movements of his heart were natural.

Percussion, pressure and deep inspirations excited very little cough.

*Examination of the Abdomen.*—No enlargement of the spleen or liver; no tenderness in the region of the former, but decided soreness in the latter, extending into the epigastrium. General soreness on pressure over the abdomen, but especially across it, in a zone passing a little below the umbilicus. Soreness in both iliac regions, extending upwards and backwards, towards the hypocondriac and lumbar regions.

Slight general fulness of the abdomen—more or less pain—and considerable flatulence.

*Examination of the Spine.*—No tenderness except near the junction of the vertebræ with the sternum. No tendency to cough from pressure.

For some time preferred to lie on his left side—now lies easier on his right; walks and rides about, and thinks himself much better than he was last winter.

The recurrence of ague and fever upon this patient, through such a long term of years, is not unprecedented, as such things have happened before in the alluvial valleys of the West; but the transition which occurred in the autumn of 1838, from misamatic, to pseudo-hectic fever is a phenomenon not often seen. The tertian character and the shake of the annual attack of that autumn, seem clearly to characterize it as of malarious origin; and, moreover, he had at that time no cough nor other pulmonary difficulty, as far as we could gather from him. But at length he became affected with a double tertian or quotidian, and while this was still returning upon him, pneumonitis of a mild kind set in, and soon afterwards night sweats began, and with the chill and nocturnal fever continued for twelve or fourteen months; when in January, 1840, he seems to have had a re-inforcement of the pneumonic inflammation. Thus we have before us a series of little interruption, for eighteen months, commencing as an endemic *ague and fever* and ending as well marked *hectic*, with a transition from one to the other, in the most imperceptible manner. Still further, the fever assumed a hectic character, while the pulmonary affection was yet too recent, according to common experience, to have produced it. It appears from this case, that a miasmatic fever has not the power to ward off a hectic, notwithstanding that opinion has had its advocates. Did not indeed the existence of a chronic intermittent, at the time the pneumonitis set in, facilitate the development of hectic—a paroxysmal fever with an evening chill, *naturally*, recurring at the same hour, which in him was the hour of recurrence of the mismatic chill? Did not the two modes of constitutional mordid action, notwithstanding the difference in their origin, so much resemble each other, as to coalesce? When, in singing, we open the mouth in the *manner* it is opened in yawning, that function is apt to be performed, although the condition which naturally prompts it is not present. But we shall not speculate further on this connexion, but request such of our readers as live in regions where ague is endemic, to keep on the look out for facts having a bearing on its relations with hectic fever.

What is the morbid anatomy of the lungs of this patient? The left is sound and sustains the *onus* of respiration. The right would appear to be impervious throughout, at least in its vesicular structure. The larger bronchial ramifications, only, admit air. How long it has been thus hepatized cannot be ascertained. From the general state of feeling of that side, and the condition of his pulse, it would appear that the inflammation had nearly ceased. Has the secretion into the parenchyma, been so great as to obliterate the capillary system to such an extent, that the inflammation ceased? May not a lung continue a long time in that condition, as it often does in a state of induration, from the compression of coagulable lymph, contracting around it? We have certainly met with cases of hepatized lung, connected with mild occasional hectic fever, which have continued for several years; and we may suppose that some very chronic cases which were regarded as tubercular have been of this kind.

The laryngeal affection in this case is distinctly marked. The relaxation of all the ligaments and muscles is indicated by the looseness of structure of that organ, and the play of the os hyoides upon it; not less than by the feebleness and flatness of the voice and cough. It is not impossible that the mucous membrane of the right side is in a state of surperficial ulceration. The tinge of blood which he lately threw up perhaps came from such a spot. Whatever may be the exact pathology of this organ, it is, perhaps, sympathetic of the disease in the right lung.

Let us turn our attention to the abdominal viscera. It is worthy of note, that notwithstanding the repetition of ague for many successive years, the spleen is not enlarged; and we may here say, what should have been said before—there is no anasarcous infiltration into the extremities.

The liver is undoubtedly in a state of chronic inflammation. The tenderness under pressure, the occasional saffron color of the urine, the turbid and sallow hue of the skin, the early tendency to diarrhœa, the tenderness *in epigastrio*, and the cough with regurgitation, after a full meal, may be received as evidence of disorder of the liver. The preference for lying on the right side is ambiguous, inasmuch, as the great amount of respiratory duty, thrown upon the left lung, requires that the weight of the body should not rest on that side. But what shall be said of the bulging out of the false ribs over the liver? Was there a swelling, inflammation and abcess of that organ, some time ago, which pressed out and changed the convexity of those ribs? Is the liver at this time enlarged *upward and outward?* Is there a collection of pus in the sac of the pleura, resting upon the diaphragm? If so, there must be adhesion of the membrane above, for the protuberance is as great in a recumbent, as upright posture.

At this time, and indeed, for many months the more energetic morbid *actions* in this case, seem to be in the mucous membrane, more especially, perhaps, that of the great intestines. We did not see the discharge which the patient described as purulent, but presume they may be of that character. Are they from the liver, or from the intestinal membrane? The latter is most probable; and taken in connexion with the general abdominal tenderness, and the tenesmus, can leave little doubt of ulceration—probably in various places. Has this condition of the membrane arisen directly from the ague and fever; or is it a sympathetic effect of the liver disease; or has it come on in the way it arises in the progress of hectic fever from tubercle?

We shall conclude these off-hand pathological speculations with a few clinical remarks and suggestions.

It appears to us, that the right lung had passed through a transformation, and that although it will doubtless, at no distant time, be the seat of a revived and suppurative inflammation of a fatal kind, there was not any existing morbid action upon which remedies need to be directed, or, indeed, could be, if morbid *action* did exist. Our thoughts turned to the state of the liver, and especially the intestinal membrane, both of which, at the present moment, seem to be the seats of sub-acute inflammatory action. Although, however, we have called the action *inflammatory* we cannot regard it of such a grade, as to call for depletion. Moreover, there seem to be several objections to a depleting plan. 1st. The pulse is not hard nor frequent. 2d. An organic lesion, so extensive as that in the lungs, renders debilitating measures, to any degree, injurious. 3d. Ulceration of the bowels may be prevented by depletion, but it by no means follows that it can be cured by the same mode. Ulcers of the skin, of the vagina and uretha, of the meatus externus, of the conjunctiva, of the mouth and throat, especially when chronic, are not in general successfully treated with emollients and refrigerants, but with stimulants, astringents and escharotics, and often heal more kindly, if tonics be administered internally at the same time; wherefore should chronic ulcers of the colon and rectum be made an exception to these, and the patient subjected to a debilitating treatment? 4th. This patient for several weeks past, has been using a bitter tincture, made with whiskey, during which time there has been no aggravation of any of his symptoms, but an increase of flesh and strength.

Under these views we made the following prescription:—

1. A liquid, demulcent and gelatinous diet—in moderation.

2. The nitro-muriatic bath, to the region of the liver, epigastrium and umbilicus, every night.

3. A draught, three times a day, of ℥ij. of decoction of cinchona, and ℥ij. of lime water, mixed.

4. A pill every night, composed of two grains blue-mass, two of ipecac. and one of opium.

Are we asked, do you expect the final recovery of the patient under this or any other treatment? We answer no.

To such of our readers as may think their time lost by the perusal of this article, we beg leave to say, that the afternoon (July 29) is oppressively hot; and that, if we were writing in as pleasant weather as they will read, we should no doubt forestall them in that criticism.

